# Endothelial Cell Amplification of Regulatory T Cells Is Differentially Modified by Immunosuppressors and Intravenous Immunoglobulin

**DOI:** 10.3389/fimmu.2017.01761

**Published:** 2017-12-14

**Authors:** Julien Lion, Maren Burbach, Amy Cross, Karine Poussin, Cécile Taflin, Srini Kaveri, Alain Haziot, Denis Glotz, Nuala Mooney

**Affiliations:** ^1^U1160, Alloimmunité-Autoimmunité-Transplantation, Institut national de la santé et de la recherche médicale, Hôpital Saint Louis, Paris, France; ^2^Department of Nephrology and Transplantation, APHP, Hopital Saint Louis, Paris, France; ^3^U1138, Institut national de la santé et de la recherche médicale, Centre de Recherche des Cordeliers, Paris, France; ^4^Université Sorbonne Paris Cité, Paris, France; ^5^LabEx Transplantex, Strasbourg, France

**Keywords:** HLA, Th17, cyclosporine A, mycophenolic acid, endothelium, organ transplantation, interleukin 6, CD54

## Abstract

Immunosuppressive treatment is a prerequisite for both organ transplantation and tolerance of the allograft. However, long-term immunosuppression has been associated with a higher incidence of malignancies and infections. Immunosuppressors mainly target circulating immune cells and little is known of their “off-target” effects, such as their impact on endothelial cells (ECs). In chronic antibody-mediated rejection (AMR), the allograft endothelium is a target of damage, histologically detected as transplant glomerulopathy, and which correlates with poor graft survival. Under inflammatory conditions, EC expression of HLA class II antigens can lead to CD4^+^-T lymphocyte alloactivation and selective expansion of pro-inflammatory Th17 and pro-tolerance Treg subsets. This response can be modified and preactivation of the EC by HLA-DR antibody binding promoted a proinflammatory Th17 response. However, whether or not immunosuppressors alter EC immunogenicity has not been examined. In alloimmunized patients with AMR, cyclosporine A (CsA) and mycophenolic acid (MPA) are often combined with intravenous immunoglobulins (IVIgs). This study reports changes in the microvascular EC phenotype and function after treatment with CsA, MPA, or IVIg. Both CsA and MPA decreased HLA-DR and increased CD54 expression, whereas IVIg increased HLA-DR expression. Interleukin 6 secretion was reduced by all three immunomodulators. Preincubation of ECs with CsA or MPA limited, while IVIg amplified, Treg expansion. Because CsA, MPA, and IVIg are known for their ability to act upon leukocytes, we confirmed that ECs maintained their immunoregulatory role when allogeneic leukocytes were pretreated with CsA, MPA, or IVIg. The results reveal that individual immunosuppressors, used in the induction and maintenance of renal allograft tolerance, had direct and distinct effects on ECs. Results of experiments associating IVIg with either CsA or MPA underlined the differences observed using individual immunosuppressors. Paradoxically, CsA or MPA may increase EC mediated inflammatory responses and long-term exposure may contribute to limitation of allograft tolerance. In contrast, IVIg interaction with the endothelium may mediate some of its immunosuppressive effects through promotion of Treg expansion, contributing to the maintenance of allograft tolerance.

## Introduction

Organ transplantation is the treatment of choice for end stage renal disease, however establishing conditions for optimal and continued graft survival is a major challenge. Immunosuppressive therapy is essential for establishing allograft tolerance, which protects the graft from damage and decreases the incidence of rejection.

The maintenance of immunosuppression is key to the prevention of acute and chronic rejections. Major improvements in graft survival resulted from the introduction of drugs (such as cyclosporine A (CsA), tacrolimus, mycophenolate mofetil and sirolimus) and antibodies (such as Basiliximab). However, chronic use of immunosuppressors is also associated with unwanted side effects, including endothelial dysfunction, an increased risk of malignancy and opportunistic infection. Long-term immunosuppressive regimens often combine a calcineurin inhibitor such as CsA and an inhibitor of nucleotide synthesis such as mycophenolic acid (MPA).

The prototypic calcineurin inhibitor, CsA is a lypophilic cyclic undecapeptide that prevents T lymphocyte proliferation by inhibiting the transcription of required cytokine genes (1) and thereby restricts T lymphocyte expansion in response to alloantigen presentation. CsA inhibits calcineurin phosphatase and so prevents the nuclear translocation of NF-AT (2) and other pathways of CsA activity include activation of JNK and of p38 MAP kinases (3). However, CsA administration has been implicated in the development of vascular complications, including hypertension and thrombotic microangiopathies (4). Furthermore, CsA treatment has been reported to induce oxidative stress and apoptosis in endothelial cells (ECs) and has been associated with enhanced EC damage and dysfunction (5–7).

Mycophenolate mofetil, the prodrug of MPA, is a reversible inhibitor of inosine 5′-monophosphate dehydrogenase (IMPDH). IMPDH is required for *de novo* purine biosynthesis and is responsible for the formation of guanine nucleotides from IMP to xanthosine 5′-monophosphate (8). Inhibition of IMPDH depletes the guanine nucleotide pool, decreases DNA synthesis and thereby reduces lymphocyte proliferation (8, 9). MPA also affects differentiation and maturation of professional antigen presenting cells including dendritic cells and B lymphocytes. MPA treatment reduces dendritic cell activation of T lymphocytes *via* the indirect pathway of antigen presentation and inhibits antibody production by lowering the number of antibody-secreting B lineage cells; this occurs without altering the expression of HLA molecules on B lymphocytes (10). Lastly, IMPDH is involved in the synthesis of membrane glycoproteins and inhibition of IMPDH alters the expression of adhesion molecules.

Although not an immunosuppressor, polyclonal intravenous immunoglobulins (IVIgs) are used to treat allosensitized transplant patients and particularly those with high levels of circulating donor specific antibodies. In contrast to the limited experience with polyclonal IVIg in transplantation, IVIg have been widely used in the treatment of autoimmune diseases (11, 12) and the efficiency of IVIg in various pathologies, including antibody-mediated rejection (AMR), has been recently reviewed (13). IVIgs are pooled preparations of immunoglobulins isolated from plasma obtained from more than 1,000 healthy donors per batch. IVIg is frequently used in the treatment of various autoimmune and immune-mediated inflammatory conditions (e.g., idiopathic thrombocytopenic purura, Kawasaki disease, dermatomyositis, systemic lupus erythematosus, and Sjogren’s syndrome). IVIg has been successfully used for the densensitization of renal and heart transplant patients with alloantibodies (14–16). Multiple and mutually non-exclusive mechanisms have been proposed to explain the benefits of IVIg including: effects on B and T lymphocytes, macrophages, dendritic cells, and natural killer cells (17–23). IVIg treatment has been reported to regulate activation and induction of Treg, to enhance Treg function by increased expression of the transcription factor FoxP3, by production of the cytokines transforming growth factor β and interleukin 10 (IL-10), and by cytotoxic T lymphocyte antigen expression (24–27).

The activity of immunosuppressors has been extensively studied in circulating immune cells, particularly T lymphocytes and professional antigen presenting cells (such as dendritic cells), because of the crucial role of these cells in the maintenance of tolerance. However, studies have largely neglected to assess the impact of immunosuppressors on EC function. Intragraft microvascular ECs form the barrier between the allograft and the recipient, and thus are in direct contact with the host immune system and the administered immunosuppressors. In the context of chronic AMR, the importance of ECs in allograft tolerance is underlined by the association between endothelial lesions and a heightened level of expression of EC activation-associated transcripts. Microvascular inflammation is also a histological indicator of AMR (28, 29).

Allograft microvascular ECs express HLA class II antigens in the steady-state and expression is highly increased under inflammatory conditions (30, 31), allowing CD4^+^-T lymphocyte activation by these cells (32). It has been reported that incubation of ECs with CsA reduced MHC class II-mediated presentation of antigen by altering intracellular antigen processing (33, 34). Regarding MPA, reduced dendritic cell expression of CD80, CD83, CD86, and CD205 has been reported, whereas HLA-DR expression was unaltered (10). In contrast, MPA treatment was associated with a dose-dependent decrease in HLA-DR expression in B lymphocytes and the same trend was observed in T cells (9).

Our previous studies revealed that HLA-DR expressing ECs regulate the allogeneic CD4^+^-T lymphocyte response, selectively and simultaneously promoting an IL-6/STAT-3-dependent proinflammatory Th17 response and a contact and CD54-dependent expansion of functionally suppressive CD4^+^CD45RA^−^HLA-DR^+^FoxP3^bright^ Treg (35). Studies in organ transplantation have conferred conflicting roles on Treg and Th17 (2) and several reports suggest that the intragraft localization of Treg is associated with decreased rejection and improved graft survival, while Th17 have been associated with promoting rejection in renal transplantation (36, 37). In humans, correlations between the proportion of Tregs within allografts and graft survival have been observed (38–40) leading to several ongoing trials of Treg infusion following organ transplantation (41). We have reported that EC allogenicity can be regulated by environmental factors. For example, inhibition of IL-6 binding promoted the Treg response whereas binding of alloantibody directed against endothelial HLA class II antigens selectively increased IL-6 secretion and the Th17 response (35, 42).

Regarding adhesion molecule expression by ECs, studies of the activity of MPA in macrovascular human umbilical vein or aortic ECs have shown conflicting results. MPA increased VCAM-1 (43), decreased ICAM-1 and VCAM-1 (44, 45), or had no effect on ICAM-1 expression (43) in TNF-α-stimulated ECs. The increased expression of VCAM-1 appeared to result from the increased stability of mRNAs (43). Olejarz et al. showed that the reduction of ICAM-1 was due to inhibition of a ROS-dependent MAP kinase, which regulates NF-κB activation and nuclear translocation (45).

Despite the many studies of immunosuppressor activity in leukocytes, their effect on endothelial allogenicity has not been addressed. We have investigated the impact of CsA, MPA, and IVIg on ECs in an inflammatory environment. The immunosuppressors MPA and CsA induced phenotypic changes, some of which were associated with altered mRNA expression. Functional studies revealed altered immunogenicity of ECs resulting in a predominantly proinflammatory CD4^+^-T cell response. In contrast, IVIg induced mRNA and phenotypic changes which were associated with a higher Treg response. Importantly immunosuppressor-promoted proinflammatory, and IVIg-promoted regulatory responses were maintained after exposure of allogeneic leukocytes to immunosuppressors suggesting that ECs could be active in systemic immunosuppression. Moreover, IVIg retained its activity on ECs when used in combination with MPA or CsA. These data contribute to understanding the effects of immunosuppressors on the endothelium and may also expose a hitherto unknown regulatory pathway initiated by IVIg interaction with ECs.

## Materials and Methods

### Cells Lines and Culture Reagents

The HMEC-1 cell line was cultured as previously described and used between passages 8 and 18 (26). Coculture experiments were carried out with ECs and non-HLA matched PBMCs, as reported (28). PBMCs were isolated from healthy donor blood samples (obtained in accordance with institutional regulations from the Etablissement Français du Sang, Paris, France) by Ficoll density gradient separation (Eurobio, Les Ulis, France).

### ECs Pretreatment with Immunosuppressors

Endothelial cells were cultured with interferon γ (IFN-γ) (200 IU/ml for 3 days; R&D Systems, Minneapolis, MN, USA) in tissue culture flasks and incubated, where indicated, with immunosuppressors: MPA (Sigma-Aldrich), CsA (Sandimmun^®^, Novartis), or with IVIg (Privigen^®^, CSL Behring) at the indicated concentrations. Parallel cultures were carried out with the relevant diluent before phenotypic, qPCR and functional assays. Methanol and ethanol were used for suspension of MPA and of CsA, respectively. Polyclonal IVIg was diluted in tissue culture medium. In certain experiments cells were pretreated with combinations of either CsA and IVIg or MPA and IVIg at the indicated concentrations.

### EC Apoptosis Assay

Cells were seeded at a density of 20,000 cells/cm^2^ and treated with IFN-γ and immunosuppressors as described above. After 3 days of treatment, apoptosis was calculated by flow cytometry using PE Annexin V apoptosis detection kit from BD Pharmingen (BD Biosciences) according to the manufacturer’s instructions.

### Real-time Polymerase Chain Reaction Analysis

CD54, HLA-DR, IL-6 and Glyceraldehyde-3-phosphate dehydrogenase (GADPH) mRNAs were assayed using a fluorescence-based real-time PCR. After 3 days of treatment with immunomodulators, total RNA was isolated from ECs using the TRI Reagent (Ambion, Applied Biosystems, Thermo Fischer Scientific) protocol. RNA was quantified using a spectrophotometer (ND-1000; Nanodrop), and converted to cDNA (1 µg RNA/reaction) by reverse transcription (RT) using the SuperScript III First-Strand Synthesis System for RT-PCR (Invitrogen Life Technologies). Real-time PCR was performed with ViiA 7 Real-Time PCR System (Applied Biosystems, Thermo Fischer Scientific) and TaqMan gene Expression Assay (Applied Biosystems, Thermo Fischer Scientific). The primers and probe sets used for this study were: IL-6 (Hs00174131_m1), CD54 (Hs00164932_m1), HLA-DR (Hs00219575_m1), and GAPDH (Hs027558991_g1). Threshold cycles (Ct) were determined as the mean of duplicate determinations. The differences in relative abundances of mRNA were calculated as 2^−Δ(ΔCt)^.

### Allostimulation Assays

After incubation of ECs with IFN-γ in the presence or absence of individual or combinations of immunosuppressors, they were washed and incubated in fresh medium overnight. Cells were then irradiated (20 Gy) and cocultured with PBMCs at a ratio 1:1 for 7 days as described (42). We have previously confirmed that the irradiation step did not prevent cytokine secretion within the following 3 days (42). In indicated experiments, PBMCs from healthy donors were incubated in the presence of immunosuppressors for 24 h before coculture. The supernatants of cocultures were collected after 72 h for cytokine measurement. At the end of the coculture, PBMCs were stimulated by phorbol-12-myristate-13-acetate 50 ng/ml, and ionomycin 1 µM (Cell Signaling Technology) in the presence of GolgiStop (BD Biosciences) for 4 h before labeling cells to detect T lymphocytes expressing intracellular IL-17 (CD3^+^CD8^−^IL-17^+^) or IFN-γ (CD3^+^CD8^−^IFN-γ^+^) by flow cytometry. Carboxyfluorescein succinimidyl ester-labeled PBMCs were used to determine proliferation of Treg (CD4^+^CD45RA^−^FoxP3^bright^) and Tmem (CD4^+^CD45RA^−^FoxP3^low^) subpopulations.

### Antibodies and Flow Cytometry

For phenotypic analysis of CD4^+^-T, the following antibodies were used: CD4 PE (Clone RPA-T4), IFN-γ FITC (Clone B27), HLA-DR allophycocyanin (APC) (clone G46-6) (BD Pharmingen; BD Biosciences), CD3 PerCP (clone SK7) (Becton Dickinson, Franklin Lakes, NJ, USA); CD4 PB (Clone RPA-T4), CD8 PB (Clone RPA-T8), CD45RA PE/Cy7 (clone H100), CD25 PE (clone M-A251), CD127 PerCP/Cy5.5 (clone A019D5), CD185 APC/Cy7 (clone J252D4) (Biolegend); and CD54 Pacific Blue (clone HCD54), IL-17 efluor660 (eBioscience). Intracellular staining of FoxP3 was carried out with the anti-Human Foxp3 Staining Set APC (clone 236A/E7) (eBioscience). Flow cytometry was carried out on a FACS Canto II (BD Biosciences).

### IL-6 Detection by Enzyme-linked Immunosorbent Assay

Assessment of IL-6 was carried out in supernatants of EC cultures or cocultures with PBMC, using an enzyme-linked immunosorbent assay detection kit from BD Biosciences, and according to the manufacturer’s protocol. All samples were assayed in duplicate.

### Statistical Analysis

Statistical analyses were performed using the GraphPad Prism (GraphPad Software, La Jolla, CA, USA). The statistical significance of the data was determined using the indicated tests (*p < 0.05, **p < 0.01, ***p < 0.001, and ****p < 0.0001).

## Results

### Immunosuppressive Treatments Modify the Phenotype of Microvascular ECs

Cell-surface expression of HLA-DR and CD54 molecules is required for amplification of Tregs, and expansion of the Th17 subset is dependent on endothelial secretion of IL-6 (35, 42). We therefore assessed the phenotypes of EC after treatment with immunosuppressors.

**Figure 1:**
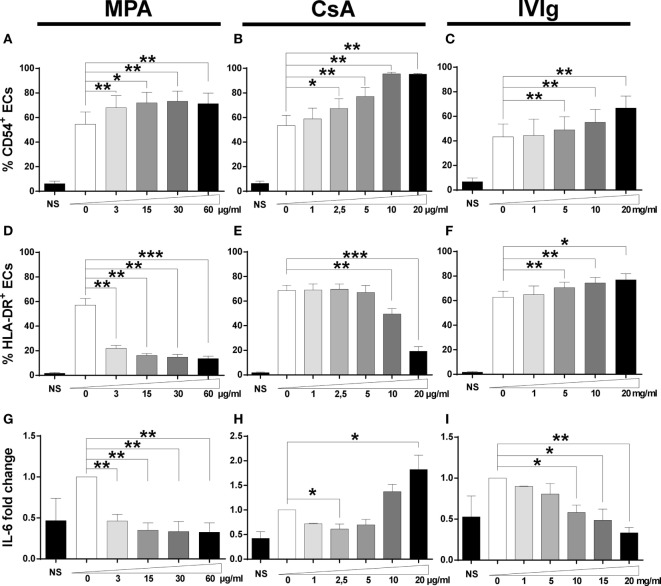
Treatment with mycophenolic acid (MPA), cyclosporine A (CsA), or intravenous immunoglobulin (IVIg) modifies endothelial cell (EC) phenotype. The phenotype of ECs was studied after 3 days of incubation with interferon γ and MPA, CsA, or IVIg at the indicated doses. NS represents EC incubated in the absence of IFN-γ, immunosuppressors, or IVIg. Control values for ECs incubated with the vehicle solutions (methanol, ethanol, or medium for suspension of MPA, CsA, or IVIg, respectively) are represented as 0. The percentage of CD54 expressing cells is shown after treatment of ECs with MPA [**(A)**, *n* = 5], CsA [**(B)**, *n* = 5], or IVIg [**(C)**, *n* = 6]. [**(D)**, *n* = 5], [**(E)**, *n* = 5], and [**(F)**, *n* = 6] show the percentage of HLA-DR expressing cells after treatment of ECs with MPA, CsA, and IVIg. [**(G)**, *n* = 5], [**(H)**, *n* = 5], and [**(I)**, *n* = 3] show the effect on interleukin (IL)-6 secretion after treatment of ECs with MPA, CsA, and IVIg, respectively. The IL-6 secretion by ECs is expressed as fold change of the level produced by cells incubated with vehicle alone. The mean ± SEM values (*p < 0.05, **p < 0.01, and ***p < 0.001, paired *t*-test) are shown.

Following incubation with MPA, an increased proportion of CD54 expressing cells and of CD54 expression was observed over a range of concentrations (Figure 1A; Figures S1 and S2 in Supplementary Material). Similarly to MPA, pretreatment with CsA increased the percentage of CD54 expressing ECs and their level of expression at most concentrations tested (Figure 1B; Figures S1 and S2 in Supplementary Material). In common with the immunosuppressors, IVIg treatment also led to an increase in the proportion of CD54 expressing ECs and in the level of expression (at concentrations ranging from 5 to 20 mg/ml) (Figure 1C; Figures S1 and S2 in Supplementary Material).

Expression of HLA-DR was reduced by MPA both in terms of the percentage of cells and the intensity of expression at all concentrations tested (Figure 1D; Figures S1 and S2 in Supplementary Material). While CsA also decreased HLA-DR expression, this was only observed at the highest concentrations tested (Figure 1E; Figure S2 in Supplementary Material). The effect of MPA on HLA-DR expression was much more marked than that of CsA and even the lowest concentration tested (3 µg/ml) reduced HLA-DR expression to 57.1% of the control level (*p* < 0.05). Finally, if MPA was added after stimulation of ECs with IFN-γ, the decrease of HLA-DR required an extended exposure to MPA (data not shown). In a previous study, the reduction of HLA-DR by MPA resulted from inhibition of guanosine synthesis, leading to an inability to transcribe mRNA and to synthesize new proteins (10).

In contrast to MPA and CsA, EC incubation with IVIg resulted in a modest but significant dose-dependent increase in the proportion of HLA-DR expressing ECs (Figure 1F; Figure S2 in Supplementary Material).

Our previous study of the signaling required for EC polarization of Th17 cells identified the role of IL-6. Both MPA and IVIg significantly reduced EC secretion of IL-6 in a dose-dependent manner (Figures 1G,I). The reduction of IL-6 was approximately 50% after cell incubation with the lowest concentration of MPA tested.

In contrast to MPA and IVIg, incubation with CsA led to a decrease in IL-6 secretion in the presence of 2.5 µg/ml but an increase at higher concentrations (10−20 µg/ml). The increased detection of IL-6 at high levels of CsA may be due to cell stress resulting in the release of stored IL-6. This is likely to be the case as detection of apoptosis after treatment of ECs with CsA, MPA, or IVIg revealed substantial apoptosis only after incubation with a high concentration of CsA (Figure S3 in Supplementary Material).

**Figure 2:**
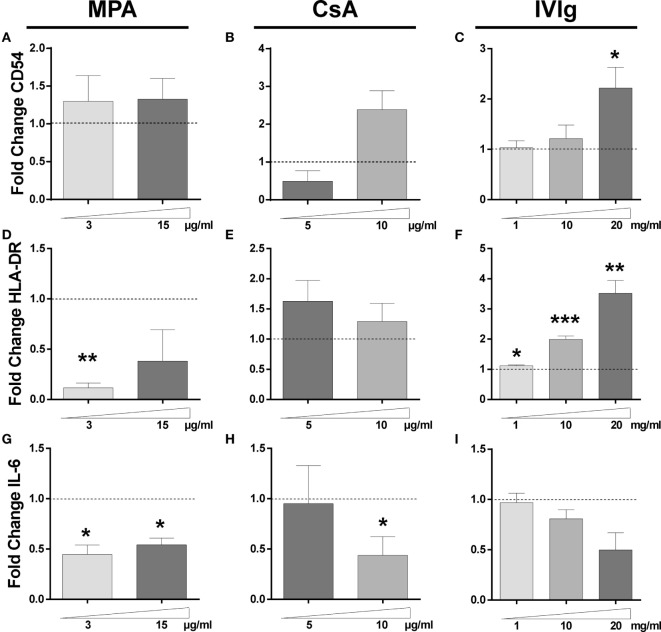
Impact of Immunomodulators on endothelial cells (ECs) gene expression. The transcription of CD54, HLA-DR, and interleukin (IL)-6 genes in ECs was examined by qRT-PCR after 3 days of incubation with interferon γ and mycophenolic acid (MPA), cyclosporine A (CsA), or intravenous immunoglobulin (IVIg) at the indicated doses. Control values for ECs incubated with the vehicle solutions. The results are expressed as fold change of GAPDH gene expression. The relative transcription level of CD54 is shown after treatment of ECs with MPA [**(A)**, *n* = 3], CsA [**(B)**, *n* = 4], or IVIg [**(C)**, *n* = 5]. [**(D)**, *n* = 3], [**(E)**, *n* = 4], and [**(F)**, *n* = 5] show the transcription levels of HLA-DR gene after treatment of ECs with MPA, CsA, and IVIg. [**(G)**, *n* = 3], [**(H)**, *n* = 4], and [**(I)**, *n* = 4] show the effect on IL-6 transcription after treatment of ECs with MPA, CsA, and IVIg, respectively. For all graphs, the mean ± SEM are indicated (*p < 0.05, **p < 0.01, and ***p < 0.001, paired *t*-test).

We next determined whether the changes in protein expression were related to modifications of mRNA expression. While MPA did not alter mRNA expression of CD54 (Figure 2A), both CsA and IVIg increased expression and this was particularly marked at high concentrations (Figures 2B,C). In contrast, mRNA levels of HLA-DR were strongly reduced by MPA (Figure 2D) [in agreement with the results of a previous study of MPA in human dendritic cells and B lymphocytes (10)]. However, HLA-DR mRNA levels were strongly increased by IVIg and not significantly altered by CsA (Figures 2E,F). When IL-6 expression was assessed, both MPA and IVIg reduced mRNA expression in agreement with the protein levels (Figures 1G,I and 2G,I) whereas CsA decreased mRNA expression at the highest concentration tested (Figure 2H). It is possible that 10µg/ml CsA activates cell stress or death (Figure S3 in Supplementary Material) resulting in inhibition of IL-6 mRNA production and simultaneous release of IL-6 stores.

The increase in CD54 protein therefore corresponds with the increase in mRNA, this is also the case for the reduced HLA-DR expression observed with MPA and the increase observed with IVIg in addition to the decrease in IL-6 induced by either MPA or IVIg. However, modifications of mRNA expression by CsA corresponded less well to the changes in protein expression. Together these data indicate that the three immunomodulators differentially alter the protein and mRNA expression of CD54, HLA-DR, and IL-6 by ECs.

### MPA, CsA, and IVIg Modify IL-6 Secretion in Cocultures of EC and PBMC

**Figure 3:**
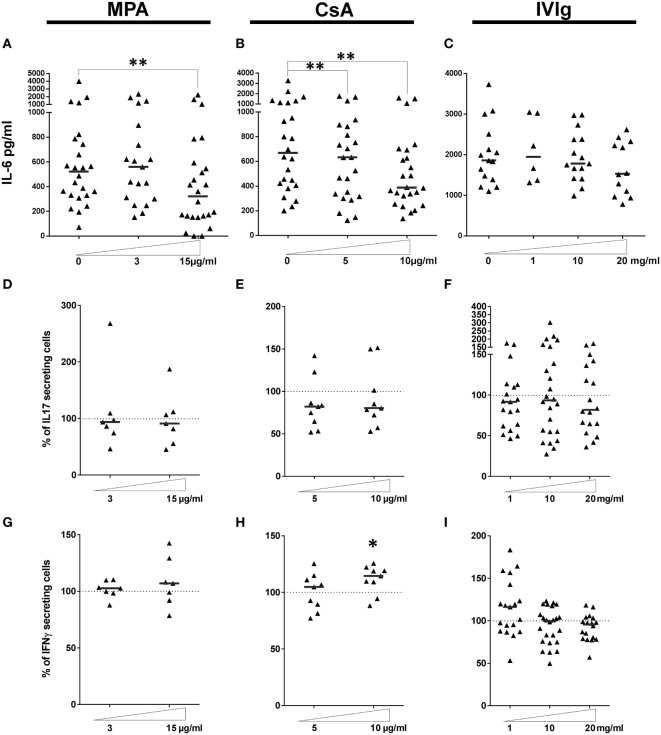
Effect of endothelial cell (EC) incubation with mycophenolic acid (MPA), cyclosporine A (CsA), and intravenous immunoglobulin (IVIg) on the proinflammatory activity of ECs. IL-6 secretion was quantified in the supernatants of cocultures where ECs was treated with MPA [**(A)**, *n* > 19 donors], CsA [**(B)**, *n* = 24 donors] or IVIg [**(C)**, *n* > 6 donors]. IL-6 production by ECs incubated with vehicle alone is represented as 0. **(D–F)** show the expansion of the Th17 subset after a 7 day coculture of PBMC with EC pretreated with interferon γ (IFN-γ) and the indicated dose of MPA [**(D)**, *n* = 7 donors], CsA [**(E)**, *n* = 9 donors], or IVIg [**(F)**, *n* > 18 donors]. Expansion of the Th1 subset under the same conditions is shown in [**(G)**, *n* > 7 donors], [**(H)**, *n* = 9 donors], and [**(I)**, *n* > 18 donors]. Results are expressed as the relative percentage of the control values (ECs treated with vehicle alone, represented by dotted lines). Thick, horizontal lines represent median values in all the cocultures (*p < 0.05, **p < 0.01: two-tailed Wilcoxon paired test).

Because the immunosuppressors and IVIg directly influenced IL-6 secretion by EC we next examined whether EC treatment with immunomodulators modified IL-6 production in EC–PBMC cocultures, since it is this context that Th17 polarization by EC is observed. As shown in Figure 3, MPA treatment reduced IL-6 in cocultures by approximately 50% at the highest concentration tested (Figure 3A). Concerning CsA, a dose dependent reduction of IL-6 secretion was also noted (Figure 3B). In contrast with the significant and dose-dependent reduction in IL-6 observed when EC alone were pretreated with IVIg (Figure 3C), IL-6 secretion was only slightly decreased in the setting of a coculture of IVIg pretreated EC and PBMC.

### Immunosuppressive Treatments Do Not Alter the Differentiation of the Proinflammatory Th17 Subset by ECs

Th17 cells have been both directly and indirectly implicated in allograft rejection (37, 46). Increased Th17 differentiation by HLA-DR expressing ECs was related to proliferation of IL-17^+^ memory T cells (Tmem) because inhibition of STAT-3 activation or of the IL-6 receptor decreased Th17 in association with decreased Tmem proliferation (35). We determined whether Tmem, or expansion of Th17 or Th1 subpopulations (Gating strategy shown in Figure S5 in Supplementary Material) are compromised by incubation of ECs with CsA, MCA or IVIg.

When the proportion and proliferation of CD4^+^CD45RA^neg^FoxP3^low^ Tmem was determined in cocultures of PBMC with ECs pretreated with MPA, CsA, or IVIg, the only difference observed was the strongly reduced proliferation of Tmem induced by MPA (Figure S4B in Supplementary Material, *p* < 0.05 compared with non-treated control). EC treatment with either CsA or IVIg did not alter the proportion or the proliferation of Tmem.

Incubation with MPA, CsA, or IVIg before coculture with PBMC did not alter Th17 expansion (Figures 3D–F). However, there was a modest, but significant, increase in the differentiation of proinflammatory CD3^+^CD8^neg^IFN-γ^+^ Th1 cells after preincubation of EC with a high concentration of CsA (10 µg/ml, *p* < 0.05, Figure 3H).

### Proliferation and Differentiation of the Treg Subpopulation Is Highly Perturbed by EC Preexposure to Immunosuppressors or to IVIg

**Figure 4:**
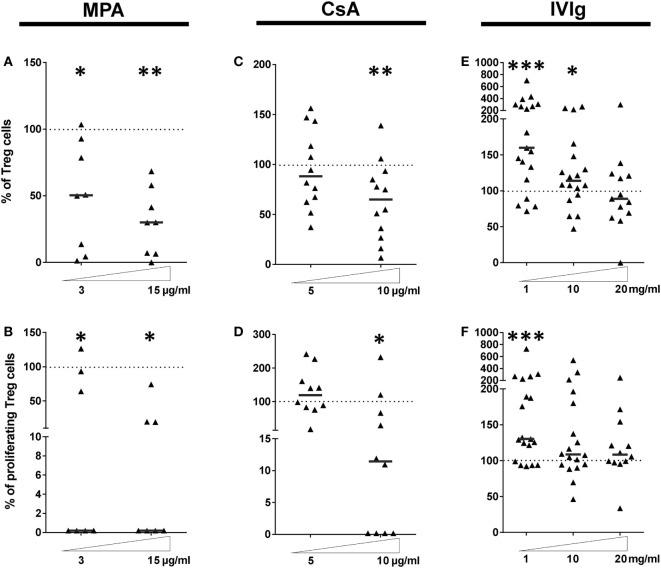
Intravenous immunoglobulin (IVIg) pretreatment of endothelial cells (ECs) induces selective amplification of regulatory T cells in contrast with the commonly used immunosupressors mycophenolic acid (MPA) and cyclosporine A (CsA). **(A,C,E)** show the proportion of Treg expanded in cocultures of PBMC with EC pretreated with interferon γ (IFN-γ) and the indicated concentrations of MPA [**(A)**, *n* = 8 donors], CsA [**(B)**, *n* = 12 donors], or IVIg [**(C)**, *n* > 13 donors]. [**(D)**, *n* = 7 donors], [**(E)**, *n* = 10 donors], and [**(F)**, *n* > 12 donors] show the proliferation of Treg after MPA, CsA, or IVIg treatment of ECs, respectively. Results are expressed as the relative percentage of the control values (ECs treated with vehicle alone and represented by dotted lines). Thick, horizontal lines show the median values in each data set (*p < 0.05, **p < 0.01, ***p < 0.001: two-tailed Wilcoxon paired test).

We next determined the effect of MPA, CsA, or IVIg on the Treg population. This population was defined as CD4^+^CD45RA^neg^FoxP3^bright^ (Gating strategy shown in Figure S5 in Supplementary Material), and are also CD25^+^ and CD127^low^. The proportion and the proliferation of Treg was strongly diminished by EC preincubation with either MPA or CsA (Figures 4A–D). The proportion of Treg was reduced to 65% of the proportion expanded by control EC (*p* < 0.01), and the percentage of proliferating Treg was reduced to 11.4% (*p* < 0.05) in the presence of CsA-treated EC. These decreases were selective for Treg as the Th17 subpopulation was unaltered by EC preincubation with CsA. Proliferation of existing Treg has been identified as a mechanism of Treg amplification; the decrease in Treg induced by MPA and CsA may therefore be due to reduced proliferation (Figures 4B,D).

These results demonstrate that EC exposure to immunosuppressors skews their orientation of the alloimmune response. CsA biased toward a proinflammatory profile by lowering Treg and modestly increasing Th1 (Figure 3E). Exposure of EC to MPA also biased toward a proinflammatory profile by reducing Treg despite an unchanged Th17 response.

Endothelial cell expression of HLA-DR was necessary and sufficient for CD4^+^-T cell proliferation and differentiation (35) and IVIg was the only immunomodulator tested that increased HLA-DR expression (respective median values of 155.1 and 113.9% with 1 and 10 mg/ml IVIg, Figure 1F). Figure 4E reveals increased Treg differentiation after preincubation of EC with IVIg. In addition, the proliferation of Treg cells was increased to a median value of 130.4% (*p* = 0.005) by IVIg-treated ECs (Figure 4F).

These data are consistent with findings from other investigators of increased Tregs after IVIg treatment in murine models of allergic airways disease or EAE (22, 26) and in the context of human autoimmune pathologies. However, in these studies, differentiation of tolerogenic DC after treatment with IVIg was identified as the mechanism of IVIg mediated Treg expansion. In the current model, only the EC were stimulated with IVIg (22) prior to coculture with PBMC from allogeneic donors.

### Exposure of PBMC to Immunosuppressors or to IVIg Does Not Prevent Altered Generation of CD4^+^-T Cell Subsets by EC Which Were Pretreated with MPA, CsA, or IVIg in Comparison with Non-Treated EC

**Figure 5:**
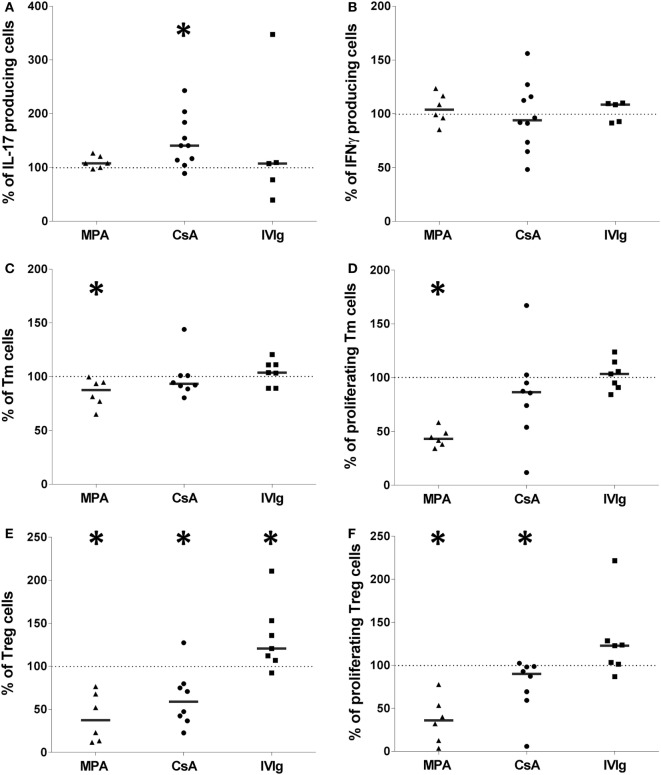
The effect of mycophenolic acid (MPA), cyclosporine A (CsA), or intravenous immunoglobulin (IVIg) on endothelial cell (EC)-mediated allogeneic CD4^+^-T cell polarization is maintained when PBMC have been exposed to immunosuppressors or IVIg. Figure shows the proportion of interleukin-17 producing cells [**(A)**, *n* > 5 donors], interferon γ (IFN-γ) producing cells [**(B)**, *n* > 5 donors], T memory cells [**(C)**, *n* > 6 donors], and their proliferation [**(D)**, *n* > 6 donors], Treg [**(E)**, *n* > 6 donors], and Treg proliferation [**(F)**, *n* > 6 donors]. Like ECs, PBMC were pretreated with MPA, CSA, or IVIg prior to coculture. In all conditions, PBMC and ECs were treated with the same immunomodulators. The concentrations used for stimulation of ECs and PBMC were 3 µg/ml for MPA, 5 µg/ml for CsA, and 1 mg/ml for IVIg. Results are expressed as the relative percentage of the control values corresponding to ECs treated with vehicle and PBMC treated by immunomodulators (represented by dotted lines). In all figures, horizontal lines show median values (*p < 0.05: two-tailed Wilcoxon paired test).

*In vivo* therapeutic use of immunomodulators would target both EC and PBMC. It was therefore important to examine whether expansion of CD4^+^-T cell populations, modified by pretreatment of ECs, were maintained after exposure of PBMC to either immunosuppressors or IVIg. The data in Figure 5 show that the effects of MPA, on EC-mediated expansion of Tmem, and Treg were conserved after exposure of PBMC to the different treatments. Pretreatment of EC and PBMC with MPA resulted in less Tmem and Treg proliferation than when only PBMC were pretreated with MPA (Figures 5C–F). The lower level of Treg proliferation was particularly marked (Figure 5F). In contrast, neither proliferation nor amplification of Th17 or Th1 was modified by MPA (Figures 5A,B). Additionally, although neither the proportion nor the proliferation of Tmem was altered by PBMC and EC exposure to CsA (Figures 5C,D), reduced Treg as well as reduced Treg proliferation, in comparison with PBMC alone, was observed (Figures 5E,F). Moreover, the proportion of Th17 cells was enhanced under these conditions (Figure 5A).

Regarding EC pretreatment with IVIg, Tmem, Th17, nor Th1 (Figures 5A–D) were significantly altered by preexposure of EC and PBMC to IVIg whereas the proportion of Treg was increased compared with Treg from pretreated PBMC alone (Figure 5E). Enhancement of Treg proliferation and differentiation by a direct action of IVIg on EC is therefore sustained when PBMC have also been exposed to IVIg prior to coculture with EC.

**Figure 6:**
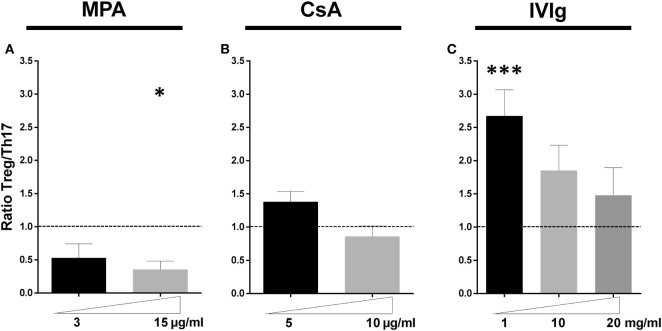
Intravenous immunoglobulin promotes a proregulatory environment in contrast to mycophenolic acid (MPA) and cyclosporine A (CsA). The ratio of the initial proportion of Treg:Th17 in the absence of MPA, CsA, or intravenous immunoglobulin (IVIg) was normalized to 1 (represented by dotted lines) for each donor and the change in this ratio after pretreatment of endothelial cell (EC) with the forementioned immunomodulators is shown. Ratio of Treg: IL-17 producing cells in cocultures of PBMC and ECs pretreated as indicated with MPA [**(A)**, *n* = 7 donors], CsA [**(B)**, *n* = 8 donors], or IVIg [**(C)**, *n* > 12 donors] is shown. For all graphs, the mean ± SEM are indicated (*p < 0.05, ***p < 0.001: two-tailed Wilcoxon paired test).

The overall equilibrium between the major CD4^+^-T cell subsets expanded by EC in this model was then determined. Figure 6 reveals the change in the ratio of Treg:Th17 relative to the initial ratio of Treg:Th17 amplified in the presence of non-treated EC. While MPA decreased the proportion of Treg:Th17, CsA did not significantly change the ratio at either concentration tested. In contrast the IVIg treatment of EC led to a two- to threefold increase in the ratio of Treg:Th17. Together these results indicate that EC are a target for either immunosuppressors or for IVIg and that the effect on ECs is maintained when PBMC have been exposed to MPA, CsA, or IVIg. These data lead to the suggestion that EC may contribute to the overall immunosuppression observed *in vivo* and as well as to the protolerance effect observed after treatment of allosensitized patients to IVIg.

### Effects of Combining CsA or MPA with IVIg

**Figure 7:**
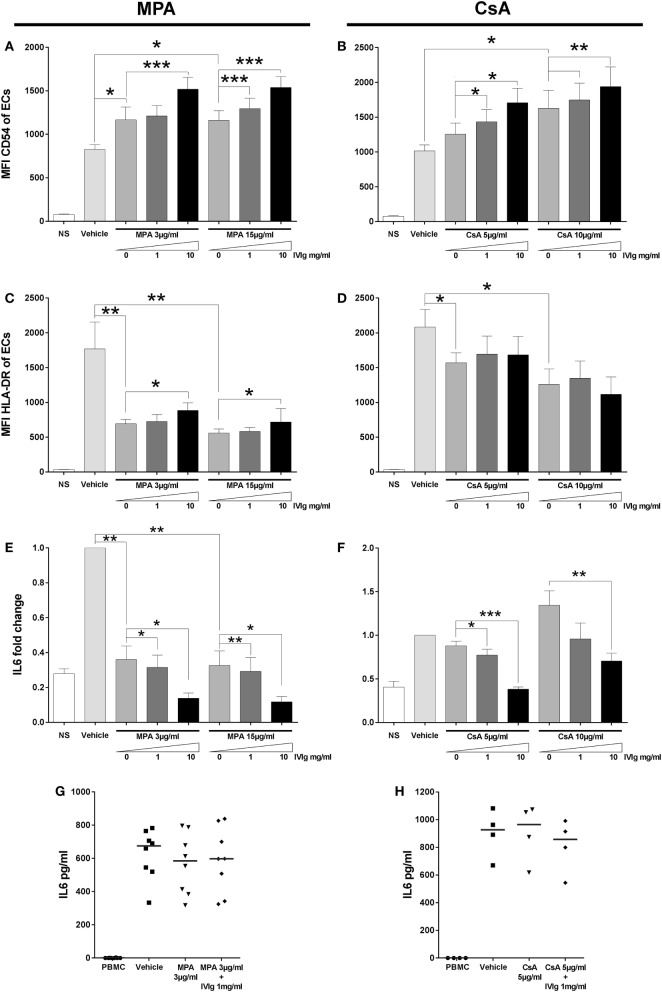
Addition of Intravenous Immunoglobulin to immunosupressors modifies their impact on endothelial cell (EC) phenotypes and interleukin (IL)-6 secretion by EC alone or in coculture. The phenotype of ECs was studied by flow cytometry after 3 days of treatment with interferon γ and the indicated combinations of immunomodulators. NS represents the “non-stimulated” condition. The control vehicle is methanol or ethanol for combinations of mycophenolic acid (MPA) with intravenous immunoglobulin (IVIg) or for combinations of cyclosporine A (CsA) with IVIg, respectively. The mean CD54 fluorescence intensity (MFI) is shown after treatment of cells with MPA and IVIg [**(A)**, *n* = 4] or with CsA and IVIg [**(B)**, *n* = 4]. [**(C)**, *n* = 5] and [**(D)**, *n* = 5] show the MFI of HLA-DR after treatment of ECs, respectively, with MPA and IVIg or CsA and IVIg. IL-6 secretion by ECs was quantified and represented as fold change of the level produced by cells incubated with vehicle alone in [**(E)**, *n* = 4] and [**(F)**, *n* = 4] where ECs was treated with MPA and IVIg or with CsA and IVIg, respectively. Finally, [**(G)**, *n* = 8] and [**(H)**, *n* = 4] show the effect on IL-6 secretion after treatment of ECs with MPA and IVIg or with CsA and IVIg, respectively, in coculture with PBMC. Horizontal columns show mean values ± SEM (*p < 0.05, **p < 0.01, and ***p < 0.001, paired *t*-test).

Mycophenolic acid and CsA belong to distinct classes of immunosuppressors, MPA targets nucleotide synthesis while CsA targets calcineurin. IVIg is distinct from both but is often used in combination with one or other. Both MPA and CsA had similar effects on EC phenotype and induced a reduction of CD4^+^CD45RA^neg^FoxP3^bright^ Treg cells. This was in marked contrast to the results of IVIg treatment. We therefore tested the outcome of EC pretreatment with immunosuppressors of different classes and IVIg: MPA and IVIg or CsA and IVIg. When ECs were pretreated with either MPA and IVIg or CsA and IVIg, the increased expression of CD54 observed with either immunosuppressor alone (confirming data in Figure 1; Figure S1 in Supplementary Material) was further enhanced by combining them with IVIg (Figures 7A,B).

Concerning HLA-DR expression, MPA alone decreased expression (confirming the data in Figure 1; Figure S1 in Supplementary Material). We also observed a higher level of HLA-DR expression in cells which had been treated with either concentration of IVIg and MPA in comparison with cells treated with MPA alone (Figure 7C). With the combination of IVIg and CsA, compensation of the loss of HLA-DR expression was not observed (Figure 7D) (unlike the combination of IVIg and MPA).

As detected for CD54 expression, associated immunomodulators had a cumulative effect on the reduction of IL-6 secretion by ECs (Figures 7E,F). When the IL-6 production was determined in cocultures of PBMC with ECs, which had been pretreated with combinations of immunosuppressors, no significant difference in the IL-6 production was observed in comparison with MPA or CsA pretreatment alone (Figures 7G,H).

## Discussion

Although endothelial lesions within the allograft have been long recognized and indeed used as a marker of allograft damage, the role of the endothelium as a mediator of the alloimmune response has recently become the object of intense study (42, 47, 48). The current study addressed whether the commonly used immunosuppressors, MPA and CsA or a regularly used treatment for alloimmunized patients, IVIg, modified endothelial allogenicity.

The results demonstrate that these immunomodulators, used to promote allograft tolerance, act directly upon the phenotype, cytokine secretion and allogeneic function of human ECs in an inflammatory environment. These data are summarized in Table 1. The immunosuppressors, CsA and MPA, promoted EC allogenicity toward a proinflammatory CD4^+^-T cell response by decreasing the amplification of Treg by ECs. In contrast, IVIg, used in the desensitization of alloimmunized transplant patients, increased the generation of a regulatory response. Modified expression of CD54 and HLA-DR, both at the mRNA and at the protein level, and altered IL-6 secretion, were observed following exposure of EC to the above immunomodulators. When MPA or CsA were combined with IVIg in order to better imitate the conditions of therapeutic use, IVIg could act to partially compensate the HLA-DR decrease mediated by MPA, while the increase in CD54 expression was further increased when IVIg was present.

**Table 1:** Summary of immunosuppressor-mediated changes in endothelial cell (EC) phenotype and in the downstream changes in allogeneic T lymphocyte polarization.

		Mycophenolic acid	Cyclosporine A	Intravenous Immunoglobulin (IVIg)
Direct changes to endothelial phenotype	Surface CD54 expression	↑	↑	↑
	Surface HLA-DR expression	↓	↓	↑
	IL-6 secretion	↓	↓↑	↓
Indirect changes to T lymphocyte polarization	% of CD4^+^ T memory cells	=	=	=
	Alloproliferation of memory T cells	↓	=	=
	IL-6 secretion	↓	↓	=
	% of Th1	=	↑	=
	% of Th17	=	=	=
	% of Treg	↓	↓	↑^a^
	% of Treg proliferation	↓	↓	↑^a^

*Changes shown are relative to non-treated controls*.

*^a^Highest increases were observed at the concentration of 1 mg/ml IVIg*.

Generation of a pro- or anti-inflammatory response was determined in a model of the interaction between microvascular EC and PBMC from non-related donors. Although the experimental set-up is an imperfect model, it recapitulates key aspects of *in vivo* interactions in the allograft microvasculature firstly because human EC activate allogeneic CD4^+^-T effector memory cells while murine ECs only activate a regulatory response and secondly because it allows monitoring of CD4^+^-T lymphocyte subpopulations relevant to the *in vivo* situation in transplant patients (32, 49).

HLA class II antigens are expressed in the steady state by human ECs and the level is strongly increased under inflammatory conditions (30, 31). Such expression can lead to activation and proliferation of allogeneic T effector memory cells (32) and to selective amplification of Th17 and Treg populations (35). Activation of proinflammatory CD4^+^-T by HLA-DR expressing ECs is further increased by EC activation with alloantibodies (42). The current study identifies new and clinically relevant regulation of EC induced pro- or anti-inflammatory responses by the immunosuppressors CsA and MPA or the immunomodulator IVIg. Moreover, the mechanism relied upon the direct activity of CsA, MPA, or IVIg on the EC.

Non-identical changes in EC phenotype were observed in the presence of CsA and MPA although both oriented the CD4^+^-T cell response toward a proinflammatory profile. For example, although HLA-DR expression was decreased by both CsA and MPA, the reduction was more drastic in the presence of MPA. This is probably due to their different targets, CsA is a prototypical calcineurin inhibitor, whereas MPA acts directly upon nucleotide synthesis. CsA has multiple effects and in an allogenic tracheal transplantation model in mice, CsA prevented CD8^+^-T cells infiltration into the graft and limited the Th1 response (50). In the current model, the effect of MPA on the cell surface expression of CD54 and HLA-DR was concordant with its effect on mRNA levels indicating that the regulation may proceed by a transcriptional mechanism. This was in contrast with CsA that did not alter mRNA levels of HLA-DR or CD54 in the same way as observed at the cell surface.

While CsA and MPA have different targets, and probably different mechanisms of altering the EC phenotype and function (suggested by the differential regulation of HLA-DR and IL-6), both enhanced the proinflammatory response, this increase may be implicated in long-term effects of CsA and MPA. IVIg differed from both CsA and MPA by increasing HLA-DR expression and by decreasing IL-6 secretion in a dose-dependent manner both at the level of the protein and of mRNA.

Although MPA and IVIg acted individually to reduce IL-6 secretion, only MPA and CsA decreased IL-6 in cocultures of EC with PBMC. The molecular interactions leading to higher IL-6 secretion by EC cocultured with allogeneic PBMC have not been identified but they may be targets of either MPA or CsA in the EC.

Intravenous immunoglobulin contrasted with both CsA and MPA by increasing the differentiation of Treg and particularly at the lowest concentration of IVIg tested. It is interesting to note that increased differentiation of Treg in response to human EC has previously been reported in the presence of Rapamycin. The mechanism was dependant on expression of the costimulatory molecule PDL1 (51). Although PDL1 is also expressed in our EC model, it was unaltered by exposure to CsA, MPA, or IVIg (data not shown). Moreover, in a previous study, we have not observed a role for PDL1 in Treg expansion (35).

The loss of the capacity for Treg differentiation by ECs exposed to CsA or MPA is striking. This may result from the decreased expression of HLA-DR, we have previously reported that HLA-DR expression is necessary and sufficient for T cell proliferation in this model and that Treg expansion relied upon proliferation (35). The importance of Treg in the transplant setting is well documented. In rodent models, Tregs significantly prolong the survival of skin (52, 53) and heart (54, 55) allografts. Correlations between the proportion of Tregs within allografts and graft survival have been observed in humans (38, 40).

The current study employed immunosuppressors and IVIg at concentrations that are close to the estimated circulating levels in transplanted patients. However, this is an approximation and even in the *in vivo* setting, exact concentrations within the graft microvasculature may not reflect those in the circulation. This may be a reason for the preferential amplification of Treg at the lower concentrations of IVIg tested. This study primarily addressed the role of the individual effects of MPA, CsA, and IVIg although these are most often administered in combinations or with corticosteroids. It will now be necessary to fully identify the pathways activated by IVIg, CsA, or MPA in ECs individually and in association.

Results of the experiments with combinations of either MPA and IVIg or CsA and IVIg underline the potential differences in their mechanisms. IVIg associated with MPA somewhat compensated the effect of MPA on HLA-DR expression, although the effect of MPA was dominant. When CsA was tested in combination with IVIg, the reduction of HLA-DR expression with CsA alone was not compensated. However, this reduction was less than that obtained with MPA alone and this could explain the lack of visible effect of IVIg.

Regarding IL-6 production, the interaction between EC and PBMC increases IL-6 production by, as yet unidentified interactions (42). The effect of either MPA or CsA on IL-6 production by ECs alone was amplified by the addition of IVIg although this effect was not visible following EC interactions with PBMC. This may be due to the low concentrations of MPA and CsA tested in combination with IVIg. Together, these results also suggest that the influence of IVIg on CsA treatment has different consequences than observed with MPA and highlight the different mechanisms of regulation of HLA-DR expression induced by CsA or MPA treatment of ECs.

Finally, these data underline the possible long-term effects of CsA and MPA in promoting EC induced proinflammatory responses. This is in clear contrast to the potential role of IVIg in boosting the EC induced regulatory response.

## Author Contributions

JL, DG, and NM designed the study and analyzed and interpreted data. JL, MB, AC, and KP acquired, analyzed, and interpreted data. AH, CT, and SK contributed to the study design. JL and NM wrote the manuscript. All authors approved the submitted version.

## Conflict of Interest Statement

CSL-Behring contributed to the funding of this study.
