# Supplementary material for: Endothelial Cell Amplification of Regulatory T Cells Is Differentially Modified by Immunosuppressors and Intravenous Immunoglobulin
Source: Front Immunol. 2017 Dec 14;8:1761. doi: 10.3389/fimmu.2017.01761 (PMC5735077; doi:10.3389/fimmu.2017.01761)
Supplement: Supplementary file 1 [file Data_Sheet_1.PDF]

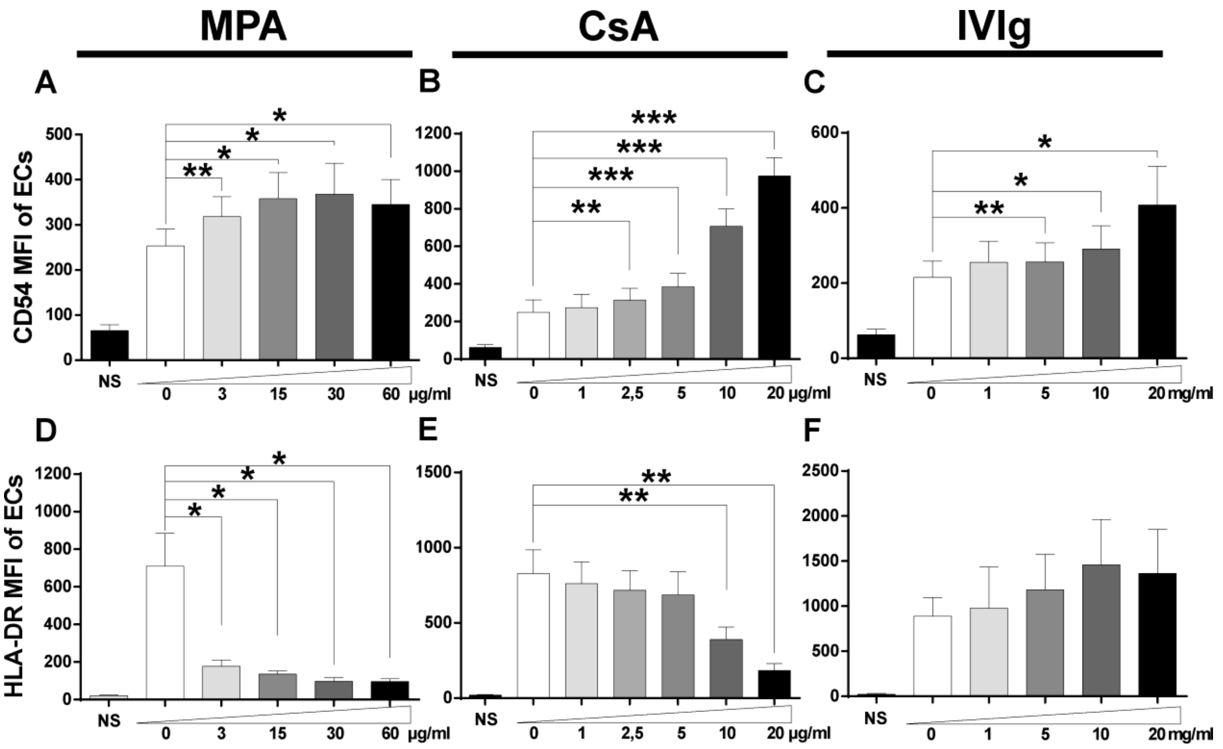


**Figure S1: The expression level of CD54 and HLA-DR on ECs was altered by MPA, CsA and IVIg.** The phenotype of ECs was studied by flow cytometry after three days of treatment with interferon γ and indicated immunomodulators. NS represents the 'non stimulated' condition. The concentrations 0 represents EC stimulation with vehicle (Methanol, ethanol or medium for suspension of MPA, CsA or IVIg respectively). The mean CD54 fluorescence intensity (MFI) is shown after treatment of cells with MPA (S1A, n=5), CsA (S1B, n=5), or IVIg (S1C, n=6). Figures S1D (n=5), S1E (n=5), and S1F (n=6) show the MFI of HLA-DR after treatment of ECs respectively with MPA, CsA and IVIg. Horizontal columns show mean values ± SEM (standard error of the mean) (*p<0.05, **p<0.01 and ***p<0.001, paired T test).
