# Supplementary material for: Endothelial Cell Amplification of Regulatory T Cells Is Differentially Modified by Immunosuppressors and Intravenous Immunoglobulin
Source: Front Immunol. 2017 Dec 14;8:1761. doi: 10.3389/fimmu.2017.01761 (PMC5735077; doi:10.3389/fimmu.2017.01761)
Supplement: Supplementary file 2 [file Data_Sheet_2.PDF]

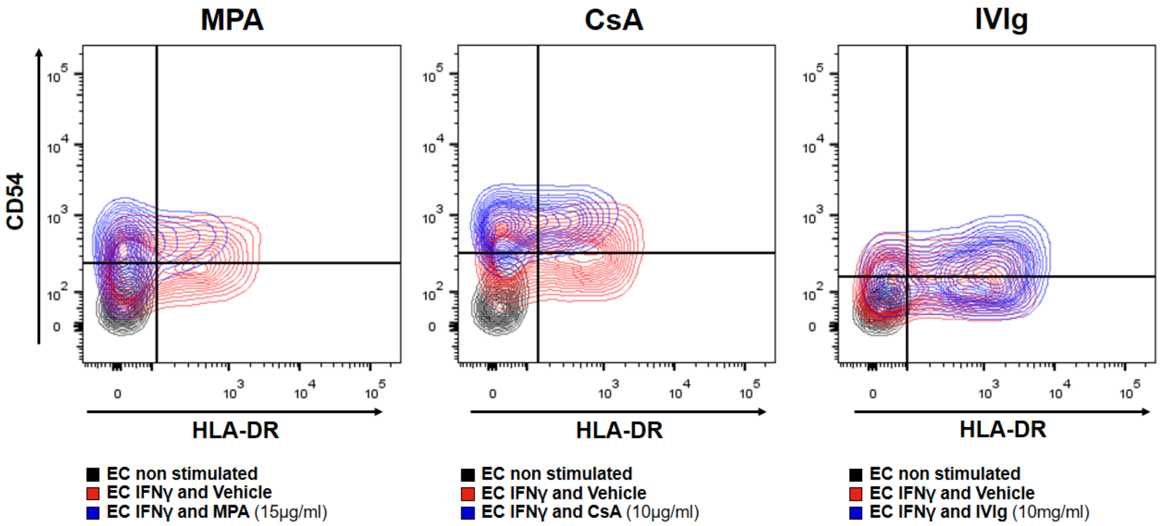

Figure S2: Representative dot plot of phenotype study after treatment of endothelial cells with Immunodulators. Dot plot represent the level of expression of HLA-DR and CD54 after three days of treatment with indicated concentration immunomodulators. An overlay of different control and conditions was performed for each immunomodulators. The level of markers expression in non-stimulated EC, in control condition with IFNγ and vehicle (Methanol, Ethanol and medium for respectively MPA, CsA and IVIg) stimulation and in “stimulated condition” where ECs was stimulated with IFNγ and immunomodulators (as indicated) in black, red and blue dot plot respectively
