# Supplementary material for: Endothelial Cell Amplification of Regulatory T Cells Is Differentially Modified by Immunosuppressors and Intravenous Immunoglobulin
Source: Front Immunol. 2017 Dec 14;8:1761. doi: 10.3389/fimmu.2017.01761 (PMC5735077; doi:10.3389/fimmu.2017.01761)
Supplement: Supplementary file 3 [file Data_Sheet_3.PDF]

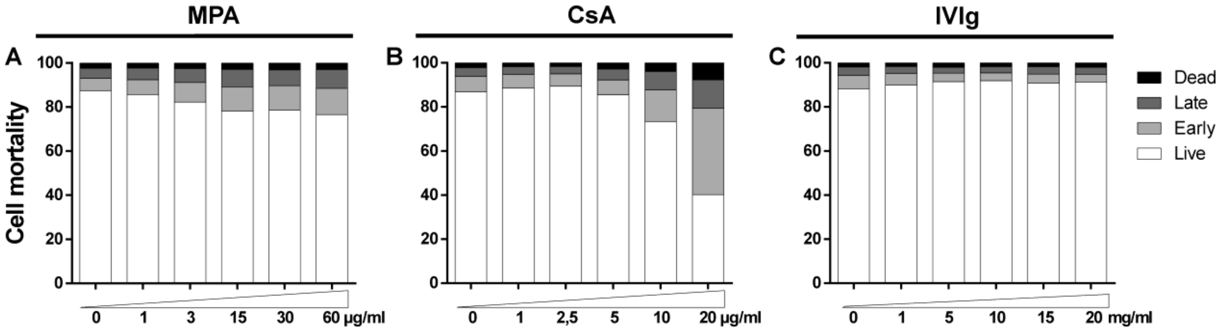

Figure S3: Treatment MCA, CsA or IVIg does not induce EC apoptosis.

Apoptosis of ECs was measured by Annexin V/7AAD staining after three days. Figure S3A, B and C show results obtained after ECs treatment with MPA, CsA and IVIg (n=3). Apoptosis of EC incubated with vehicle is indicated by the point 0 (Methanol, ethanol or medium for suspension of MPA, CsA or IVIg respectively). The percentage of live cells (Annexin V^{neg} /$7AAD^{neg}$) named "Live", cells in early apoptosis (Annexin V^{pos} /$7AAD^{neg}$) named "Early", cells in late apoptosis (Annexin V^{pos} /$7AAD^{pos}$) named "Late" and dead cells (Annexin V^{neg} /$7AAD^{pos}$) named "Dead" are represented by histograms.
