# Supplementary material for: Endothelial Cell Amplification of Regulatory T Cells Is Differentially Modified by Immunosuppressors and Intravenous Immunoglobulin
Source: Front Immunol. 2017 Dec 14;8:1761. doi: 10.3389/fimmu.2017.01761 (PMC5735077; doi:10.3389/fimmu.2017.01761)
Supplement: Supplementary file 4 [file Data_Sheet_4.PDF]

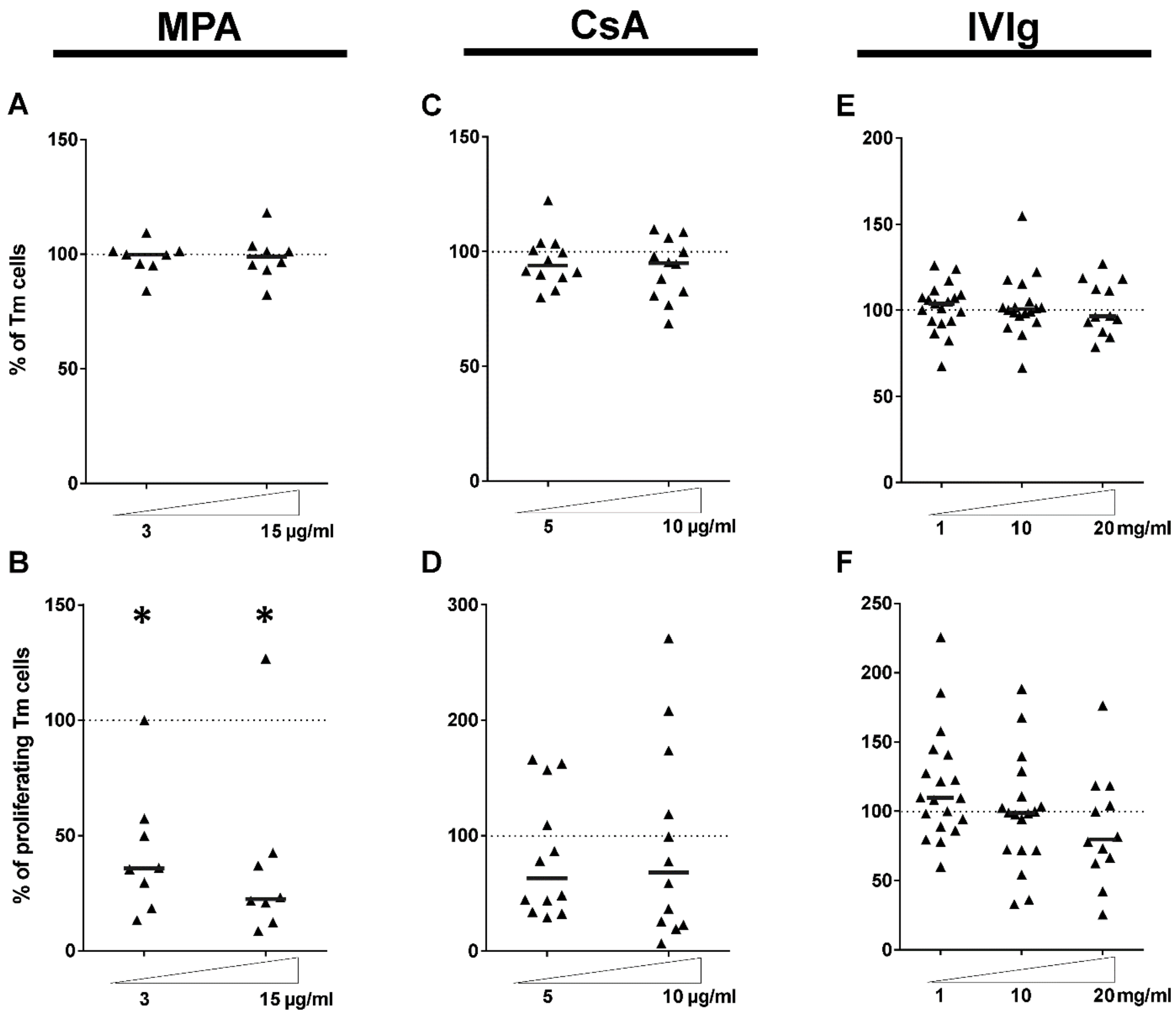

Figure S4: MPA treatment of ECs reduced proliferation of T memory cells. Figures S4A, C and E show the percentage of T memory cells (Tm) in PBMC cocultured for seven days with EC pre-treated with MPA (S4A, n=8 donors), CsA (S4C, n=12 donors) or IVIg (S4E, n>12 donors). The proliferation of T memory cells was also examined and the data obtained is shown in graphs S4B, S4D and S4F for MPA (n=8 donors), CsA (n=12 donors) or IVIg (n>12 donors) respectively. Results are expressed as the relative percentage of the control values (ECs treated with vehicle is represented by dotted lines). For all graphs, horizontal lines show median values (*p<0.05: two-tailed Wilcoxon paired test).
