# Supplementary material for: Endothelial Cell Amplification of Regulatory T Cells Is Differentially Modified by Immunosuppressors and Intravenous Immunoglobulin
Source: Front Immunol. 2017 Dec 14;8:1761. doi: 10.3389/fimmu.2017.01761 (PMC5735077; doi:10.3389/fimmu.2017.01761)
Supplement: Supplementary file 5 [file Data_Sheet_5.PDF]

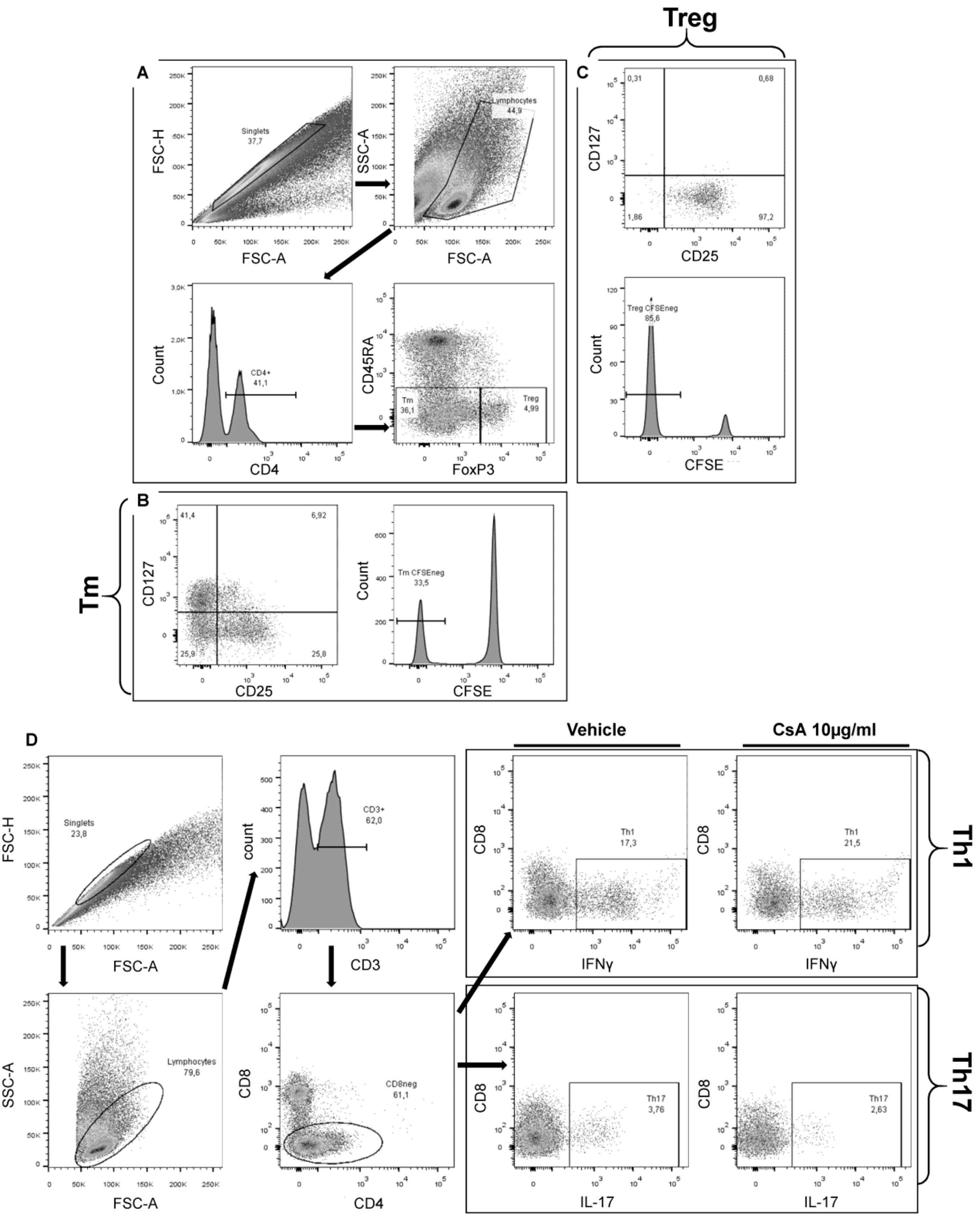

Figure S5: Gating strategy for identification of regulatory T cells, Th1 cells and Th17 cells. Figure S4A show the gating strategy for identification of $CD4^{+}CD45RA^{neg}FoxP3^{high}$ Treg cells after selection of single events. The expression of CD127 and CD25 in $CD4^{+}CD45RA^{neg}FoxP3^{low}$ Tm and in $CD4^{+}CD45RA^{neg}FoxP3^{high}$ Treg cells was examined (Figure S4B and C). The proliferation of $CD4^{+}CD45RA^{neg}FoxP3^{low}$ Tm and $CD4^{+}CD45RA^{neg}FoxP3^{high}$ Treg was assessed after staining with CFSE. Figure S4D show the gating strategy for identification of $CD3^{+}CD8^{neg}IFN\gamma^{+}$ Th1 cells and of $CD3^{+}CD8^{neg}IL\text{-}17^{+}$ Th17 cells after selection of single events. The characterisation of Th1 and Th17 population after co-culture of EC stimulated with Vehicle or CsA at 10µg/ml with PBMC was represented.
